# An effective approach for plant leaf diseases classification based on a novel DeepPlantNet deep learning model

**DOI:** 10.3389/fpls.2023.1212747

**Published:** 2023-10-11

**Authors:** Naeem Ullah, Javed Ali Khan, Sultan Almakdi, Mohammed S. Alshehri, Mimonah Al Qathrady, Nora El-Rashidy, Shaker El-Sappagh, Farman Ali

**Affiliations:** ^1^ Department of Software Engineering, University of Engineering and Technology, Taxila, Pakistan; ^2^ Department of Computer Science, Faculty of Physics, Engineering, and Computer Science, University of Hertfordshire, Hatfield, United Kingdom; ^3^ Department of Computer Science, College of Computer Science and Information System, Najran University, Najran, Saudi Arabia; ^4^ Departments of Information Systems, College of Computer Science and Information Systems, Najran University, Najran, Saudi Arabia; ^5^ Machine Learning and Information Retrieval Department, Faculty of Artificial Intelligence, Kaferelshikh University, Kafr El-Shaikh, Egypt; ^6^ Faculty of Computer Science and Engineering, Galala University, Suez, Egypt; ^7^ Information Systems Department, Faculty of Computers and Artificial Intelligence, Benha University, Banha, Egypt; ^8^ Department of Computer Science and Engineering, School of Convergence, College of Computing and Informatics, Sungkyunkwan University, Seoul, Republic of Korea

**Keywords:** artificial intelligence, deep learning, DeepPlantNet, leaf diseases, plant diseases classification

## Abstract

**Introduction:**

Recently, plant disease detection and diagnosis procedures have become a primary agricultural concern. Early detection of plant diseases enables farmers to take preventative action, stopping the disease's transmission to other plant sections. Plant diseases are a severe hazard to food safety, but because the essential infrastructure is missing in various places around the globe, quick disease diagnosis is still difficult. The plant may experience a variety of attacks, from minor damage to total devastation, depending on how severe the infections are. Thus, early detection of plant diseases is necessary to optimize output to prevent such destruction. The physical examination of plant diseases produced low accuracy, required a lot of time, and could not accurately anticipate the plant disease. Creating an automated method capable of accurately classifying to deal with these issues is vital.

**Method:**

This research proposes an efficient, novel, and lightweight DeepPlantNet deep learning (DL)-based architecture for predicting and categorizing plant leaf diseases. The proposed DeepPlantNet model comprises 28 learned layers, i.e., 25 convolutional layers (ConV) and three fully connected (FC) layers. The framework employed Leaky RelU (LReLU), batch normalization (BN), fire modules, and a mix of 3×3 and 1×1 filters, making it a novel plant disease classification framework. The Proposed DeepPlantNet model can categorize plant disease images into many classifications.

**Results:**

The proposed approach categorizes the plant diseases into the following ten groups: Apple_Black_rot (ABR), Cherry_(including_sour)_Powdery_mildew (CPM), Grape_Leaf_blight_(Isariopsis_Leaf_Spot) (GLB), Peach_Bacterial_spot (PBS), Pepper_bell_Bacterial_spot (PBBS), Potato_Early_blight (PEB), Squash_Powdery_mildew (SPM), Strawberry_Leaf_scorch (SLS), bacterial tomato spot (TBS), and maize common rust (MCR). The proposed framework achieved an average accuracy of 98.49 and 99.85in the case of eight-class and three-class classification schemes, respectively.

**Discussion:**

The experimental findings demonstrated the DeepPlantNet model's superiority to the alternatives. The proposed technique can reduce financial and agricultural output losses by quickly and effectively assisting professionals and farmers in identifying plant leaf diseases.

## 1 Introduction

The earth’s ecosystem, a geographically dispersed natural setting, includes plants undistinguishably. The planet’s habitat is growing by roughly 1.6% annually (Garg and Singh, 2023). Thus, rising demand for plants and items made from plants is increasing. A wide range of biotic stressors may impact agricultural yields, and due to decreased output levels, there may be significant financial losses. Food safety, agriculture, and nutrition are all interrelated and significantly affect people’s well-being (Food safety, 2022). Also, it has an undesirable impact on the poor and underdeveloped world, causing problems with their economies and health.

Additionally, since the world’s population is continuously growing, there is a daily rise in the demand for food. A country where farming is still a key driver of economic growth understands the need to safeguard crops from the devastation of leaf illnesses. These are the main reasons for both amount and quality losses in agricultural productivity. These losses have a negative impact on the agricultural companies’ production costs as well as their revenue margins. There aren’t enough existing quick and precise identifying tools. The nation’s nutritional security, food supply, the welfare of farmers, and way of life are all gravely threatened by illness occurrences of any kind. Due to the late discovery of plant illnesses, food poverty will worsen. In demand to successfully avoid and cure plant infections, it is crucial to identify them as soon as feasible. Several studies are being done to protect plants against diseases, and integrated pest management techniques are also being used to supplement conventional pesticides. The automatic plant disease categorization method is crucial for locating plant illnesses. Plant diseases are also significant in the decision-making and supervision procedures related to agricultural production. Recently, it has become vital to identifying plant diseases automatically. The considerable danger to the global food supply is an illness in plants, and it is difficult to identify many conditions in time. The leaves and stems of damaged plants, as well as their flowers, stalks, and fruits, will typically have noticeable lesions or markings. In general, it is possible to discern anomalies from one another by using the distinctive visual pattern that each illness or pest scenario possesses. The bulk of disease symptoms may typically be found on a plant’s leaves, which are also the primary source of information for diagnosing plant illnesses (Ebrahimi et al., 2017).

Farmers and plant pathologists have traditionally depended on their eyes to diagnose illnesses and form opinions founded on their prior knowledge. Still, this method has occasionally been wrong and misinterpreted because numerous distinct conditions initially appear to be the same. Due to the diversity of plants, different crops also exhibit diverse disease features, which adds a great deal of complexity to the classification of plant illnesses. Also, the expertise of farmers and plant pathologists must be passed on from one generation to the next. Yet, visual analysis of the leaf crown structures and color patterns remains the primary tool in conventional field screening for plant disease. Humans need time, effort, and specialized knowledge to identify plant diseases based on their experience and careful observation of the disease’s signs on plant leaves (Sankaran et al., 2010). Due to the diversity of plants, different crops also exhibit diverse disease features, which adds a great deal of complexity to the classification of plant illnesses.

Moreover, the wrong prediction of plant diseases causes the overuse of pesticides, which raises manufacturing costs. Based on these facts, creating a reliable disease identification system connected to a dedicated database is crucial to help farmers, especially young and inexperienced farmers. Researchers have developed novel approaches to identify plant diseases utilizing image processing to address these issues. The research agenda now places this at the top.

Several works have concentrated on categorizing plant illnesses using machine learning (ML). Most of the ML research has focused on classifying plant diseases using characteristics of plant leaf images, such as type (Mokhtar et al., 2015), color (Rumpf et al., 2010), and texture (Hossain et al., 2019). The three main categorization methods are K-nearest neighbors (Hossain et al., 2019), support vector machines (SVM) (Rumpf et al., 2010), and random forests (Mohana et al., 2021). Following is a list of these techniques’ main drawbacks: ML-based methods did not work well (poor performance) and could not be employed for real-time categorization. They frequently need to manually develop and extract features, which calls for the professional skills of research employees. Manual feature extraction is also time-consuming and complex. Most conventional methods use fundamental feature extractors, including hand-crafted features, shape-based feature extraction, histogram of gradients, and scale-invariant feature transformation (SIFT). These customized elements must be extracted through a complex process that takes scale-invariant to complete. After training the features, various learning algorithms—including the SVM—are used to classify different information types. The quantity of image preparation required by conventional methods may be relatively high, adding to the required effort and time. This covers image scaling, denoising using a smoothing filter like a Gaussian, and other image processing techniques. These preprocessing steps increase the processing duration of the pipeline for disease detection.

The research was motivated by the fact that despite the large variety of studies on the categorization of plant leaf diseases, there is still interest in creating high-accuracy automated systems for this purpose. Although a few studies on the categorization of plant leaf diseases have lately been offered, this area of study is still not fully investigated. The most often used plant leaf diseases classification techniques in current research are transfer learning (TL) of pre-trained DL frameworks and support vector machines (SVM). The SVM machine learning technique, however, needs more time to train with bigger datasets. The constraints in TL that cause the most worry are overfitting and negative transfer. To solve these issues in this research work, we created the DeepPlantNet model for plant leaf diseases classification.

Plant leaf diseases classification has also benefited from applying deep learning (DL) techniques with promising outcomes in recent years. DL techniques are currently being used widely in the agricultural sector for applications, including weed recognition (Yu et al., 2019), crop pest categorization (Ullah et al., 2022a), and plant illness identification (Bansal et al., 2021). One of ML’s study focuses is DL. It has primarily addressed the issues with standard ML approaches’ segmented operation (Athanikar and Badar, 2016), poor performance, long processing time, and manual feature extraction. The key benefit of DL models is their ability to extract features without the need for segmented operations while still achieving acceptable performance. Automatic feature extraction from the underlying data occurs for each object. The development of CNNs has increased the efficiency and automation of plant disease classification technology. While being quite effective at identifying the disease, typical convolutional neural network models are more expensive to compute. This necessitates the creation of a model that is effective and involves the generation of fewer parameters. In this paper, inspired by the effectiveness and success of DL, we suggested the DeepPlantNet model for eight types of plant disease classification of eight plants. To the best our knowledge it is the first study which uses different datasets and perform three types of classification tasks i.e., eight-class, six-class, and three-class classification of plant leaves. Our model contains only 28 learned layers, i.e., 25 ConV and 3 FC layers. Improved detection performance can be attained using the filter-based feature extraction method in the proposed framework. This study developed a model that would identify plant leaf diseases with enhanced accuracy and efficiency compared to the current methodologies. Most of the convolution filters employed in our model are 1×1 since 1×1 kernels have fewer parameters than 3×3 filters, ultimately decreasing the number of parameters. The suggested model can categorize photos into many classifications. The proposed approach employs a convolutional layer and leaky relu (LReLU) AF to extract the high-level characteristics from images. We categorize the diseases of eight different types of plants (grape, apple, pepper, cherry, peach, potato, strawberry, and squash) into the following eight groups: ABR, CPM, GLB, PBS, PBBS, PEB, SPM, and SLS. Furthermore, we have performed three-class classifications to classify plant diseases, including bacterial tomato spots, an early blight on potatoes, and standard corn (maize) rust. We also performed six-class classification to incorporate the healthy leaf images as well. It merges low-level features to produce abstract high-level features to uncover generalized features and attributes of sample data. The study’s primary contributions are:

- We developed an efficient DeepPlantNet model to improve plant disease classification by automatically detecting plant diseases in various phases of development in different plants.
- The suggested novel end-to-end DeepPlantNet framework automatically extracts the most discriminative characteristics for accurate plant disease classification and recognition.
- We have performed eight-class, six-class, and three-class classification experiments to classify plant diseases into eight and three types.
- We evaluated the efficacy of the suggested approach against existing cutting-edge models for identifying and classifying plant diseases.

The article is separated into the following segments: The literature review is offered in Section 2. The DeepPlantNet framework’s operation is then thoroughly discussed in Section 3. The proposed work’s performance evaluation is provided in Section 4. Whereas, section 5 discusses our method and obtained results. Lastly, the overall conclusion is presented in Section 5 at the end.

## 2 Related work

Many studies in the area of machine vision in agriculture have been conducted recently, including studies on fruit disease diagnosis (Hariharan et al., 2019), quality rating and fruit maturity classification (Zhou et al., 2021), plant pest classification (Ullah et al., 2022a), plant species classification (P’ng et al., 2019), and weed control and recognition (Dadashzadeh et al., 2020). The plant leaf diseases classification has received some recent study attention. Mainly, ML and DL techniques are used to categorize numerous groups of plant illnesses in various plants. The most current and pertinent research on automatic plant disease detection and classification are highlighted below.

In (Sahu and Pandey, 2023), the authors created a new hybrid random forest Multiclass SVM (HRF-MCSVM) framework to detect plant foliar diseases. Before classification, the picture features are segmented and preprocessed utilizing spatial fuzzy c-means to improve computation accuracy. They used a dataset from the Plant Village dataset contains photos of both healthy and sick leaves. The system’s effectiveness was then assessed using performance indicators like F-measure, accuracy, sensitivity, recall, and specificity value. In (Ratnasari et al., 2014), the authors introduced a sugarcane leaf disease identification technique using RGB pictures. Only three categories of diseases—ring spot, rust spot, and yellow spot—have undergone verification using the suggested system. While SVM was employed for classification, feature extraction used a mixture of texture and color features. Four kernel types—quadratic, polynomial, linear, and radial basis functions—were examined for the SVM classifier, with the linear kernel outperforming the others in performance. In (Rastogi et al., 2015), the authors created a machine vision-based system for detecting and classifying maple and hydrangea leaf illnesses. Leaf RGB pictures were first subjected to preprocessing, and then Euclidean distance and K-means clustering techniques were used for segmentation. The GLCM matrix, in which energy, contrast, correlation, and homogeneity have been determined, is considered for extracting features. ANN has been utilized for categorization in this study. For grading, the proportion of infection has been estimated using the disease and total leaf area. Fuzzy logic has been used to grade once the infection has been determined.

Earlier investigations used models with hand-crafted characteristics built on plant leaf shape, color, and texture to categorize plant diseases. Extreme feature engineering was used in these investigations, which mainly focused on a few disorders and were often restricted to specific contexts. Approaches of ML rely on significant preprocessing procedures, including a manual area of interest trimming, color modification, scaling, filtering, and background exclusion, for successful feature extraction since the obtained features are sensitive to the environment seen in photographs of leaves. Traditional ML approaches could only classify a few diseases from limited data because of these preprocessing techniques’ increasing complexity, and they could not scale to more significant sizes (Ahmed et al., 2022).

Current TL-based methods utilizing the PlantVillage database for leaf disease prediction have evaluated the efficacy of several DL frameworks utilizing numerous hyperparameters in demand to decrease dependency on hand-crafted features and increase categorization performance with massive datasets. The authors used the conditional generative adversarial network (C-GAN) (Abbas et al., 2021) to make artificial images of the tomato plant leaves to identify tomato disease. This model was one of these experiments. Then, using TL, a DenseNet-121 framework is trained to categorize pictures of tomato leaves into five, seven, and ten illness groupings. The authors of (Vickers, 2017) employed the CNN framework for classification tasks and the VGG network for illness localization. When contrasted with the statistics compiled by the authors (Sharma et al., 2022), this model attained a reasonable level of accuracy. In (Kumari and Singh, 2018), the authors segmented data using the VGG16 framework and classified it using the AlexNet framework. Yet the categorization accuracy of this approach is poor.

In (Chen et al., 2020), the authors used an AlexNet framework built on deep transfer learning for classification tasks. However, this approach was unable to isolate the disease-affected area. In (Yang et al., 2020) the authors described how a U-Net segmentation and ANN were used to detect illnesses in various plant types, including tomato, mango, lemon, potato, jackfruit, beans, Sabota, and bananas. Because of the KMC process’ assistance in determining the location of the disease, this technique also offers a quicker segmentation procedure. The authors proposed a method for timely plant illness identification that combines ANN with a Gabor filter for feature extraction (Kulkarni and Patil, 2012). The ANN performed the categorization procedure using texture and color data. This study revealed a significant research gap in several methods: the need for larger datasets for accurate implementation. Several mixtures of data preprocessing, including Contrast Limiting Adaptive Histogram Equalization (CLAHE) on individual RGB channels, RGB to HSV adaptation of pictures, and log transformation, were reported in (Jasrotia et al., 2023) along with a customized CNN-based Maize Plant Illness Detection framework. These trained frameworks contrasted with the CNN and SVM models that were trained without preprocessing procedures. The studies were run on the PlantVillage Maize Crop Database to gauge the efficacy of the proposed effort.

In contrast to the current methods, the authors (Joshi and Bhavsar, 2023) proposed a model to more accurately and effectively diagnose the illnesses of plant leaves. The Night shed plant leaf, obtained from the plant village dataset, was utilized for training the proposed framework and the industry-standard models AlexNet, VGG, and GoogleNet. There are nine distinct categories of illnesses as well as healthy plant leaves. The effectiveness or accomplishment of the models was assessed using a wide range of variables, including dropout, AF, batch size, and learning rate.

According to current studies, the categorization of plant diseases is primarily done using ML and DL models. However, their increasing computing complexity is hampered these methods’ main issues. There is still room for improvement in the trade-off between accuracy and computing complexity. Yet, no effort is shown to design a lightweight framework for classifying plant diseases. As the goal of our work, we have suggested a simple framework for classifying plant leaf diseases that consider these gaps.

## 3 Methodology

**Figure 1:**
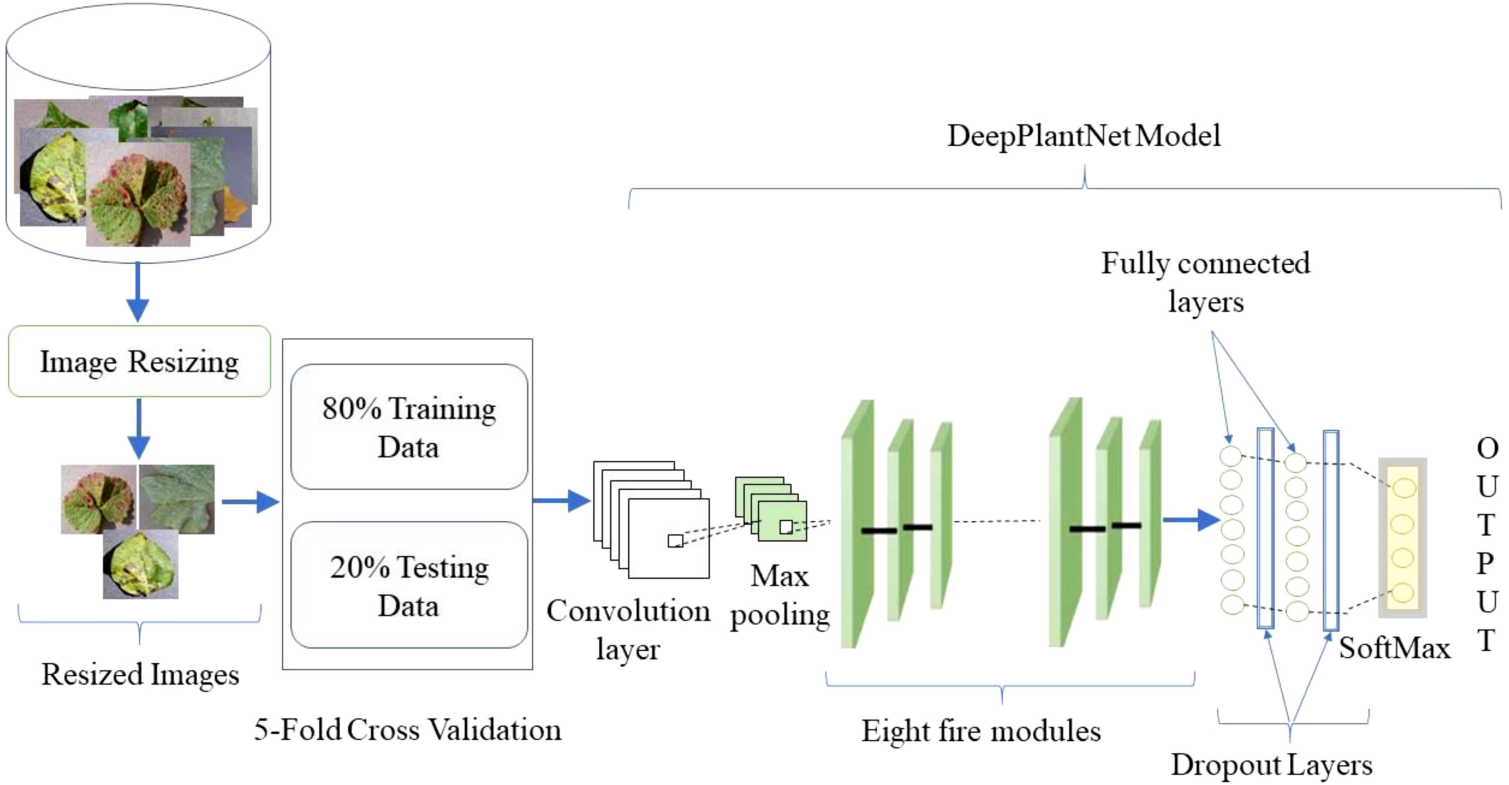
General workflow of the suggested approach.

The plant leaf diseases classification can be made utilizing digital image processing. Digital image processing has advanced thanks to DL, outpacing conventional techniques in recent years. We proposed the DeepPlantNet model for identifying plant leaf illnesses in this work. Employing plant leaf images from the publicly available database, we will locate diseases of eight different types of plants (apple, cherry, grape, peach, pepper, potato, squash, and strawberry) into the following eight groups: ABR, CPM, GLB, PBS, PBBS, PEB, SPM, and SLS. The general description of the proposed method is shown in **Figure 1**. We provided the framework with images of various sizes to put the proposed method into effect. Then, we applied some pre-processing to decrease the dimensions of the input images to 227×227 pixels to ensure uniformity and accelerate the procedure. A DeepPlantNet framework with only 25 ConV layers was developed to classify plant leaf images into eight classes. For all experiments, separate data sets are utilized for training and testing. Specifically, we used 20% of the plant leaf images for model testing and 80% of the plant disease images for training.

### 3.1 Dataset

**Figure 2:**
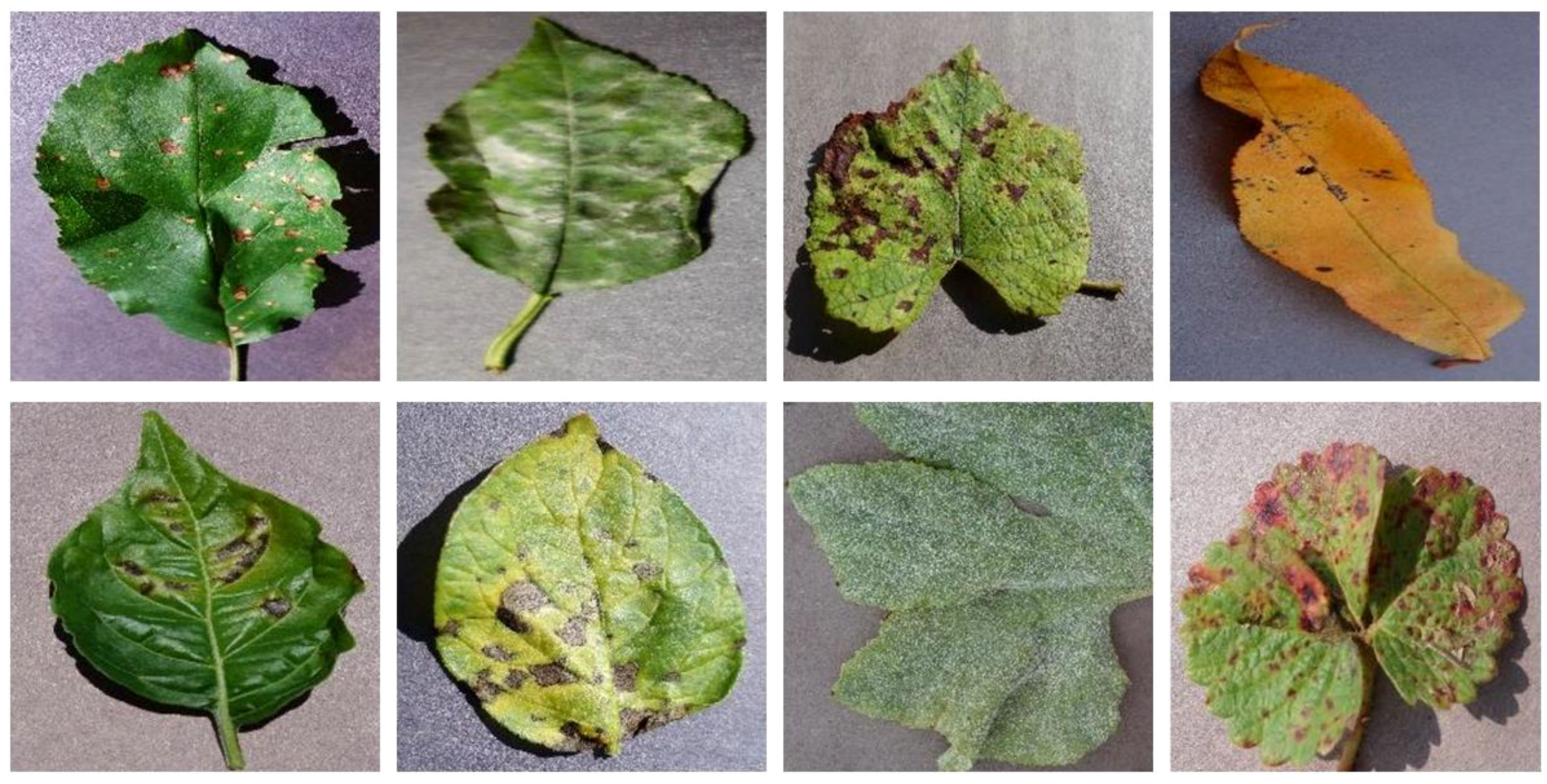
Representative samples from the dataset, the first row contains images of ABR, CPM, GLB, and PBS, whereas the second row contains images of PBBS, PEB, SPM, and SLS.

We verified the effectiveness and robustness of the DeepPlantNet model by using images from the publicly available Kaggle “PlantVillage Dataset” dataset (Dataset: https://www.kaggle.com/datasets/abdallahalidev/plantvillage-dataset). About 54,000 photos of healthy leaves and illness cases are included in the open-source PlantVillage collection, divided into 38 categories by 14 species and pathologies. Dataset images vary in lighting, angles, format, bit depth, dimensions, and size, among other factors. We have only used infected leaf images of eight species (cherry, apple, peach, grape, strawberry, pepper, squash, and potato) and considered only one significant disease of each class (species). We have utilized images of eight categories in this study, including ABR, CPM, GLB, PBS, PBBS, PEB, SPM, and SLS. We used 260 images of each type for testing and training our model in this study. Some representative samples of the dataset are given in **Figure 2**.

### 3.2 Experimental setup and evaluation

On a Computer with an Intel (R) Core (TM) i5-5200U CPU and 8GB of Memory, all the trials (experiments) were run. MATLAB R2020a was used to carry out the approach. The database’s images are fragmented into training and testing sets for all experiments. We do several experiments to see how well our suggested framework classifies plant diseases (**Table 1**).

**Table 1:** Information about the implementation system.

Sr. no	Name	Experiment parameters
1	System type	Windows 10, 64 bit
2	CPU	Intel (R) Core (TM) i5-5200U
3	RAM	8GB
4	HDD	500GB
5	Development tool	MATLAB R2020a

### 3.3 Image resizing

Images of plant leaves in the database are in a range of dimensions. We pre-processed the plant leaf images to resize down to 227×227 pixels in line with our classifier’s specifications (input image) to ensure uniformity and hasten the procedure.

### 3.4 K-fold cross validation

The K-fold, or 5-fold cross validation, is used to train our DeepPlantNet model, and the folding is carried out five times. The dataset will be divided into 5 equal parts due to the usage of K-fold cross validation, and the testing set will be modified each time. The testing will alter gradually for the third, fourth, and fifth testing sets of data as well. For the initial training, the testing set will be the first fold or first 20% of the data. Here it is important to note that 80% of the dataset will always be training data.

### 3.5 DeepPlantNet model architecture details

In this work, we proposed the DeepPlantNet model for categorizing eight different plant diseases in eight other plants. The DeepPlantNet model comprises just 28 learned layers, including 25 ConV and 3 FC layers. Our model consists of 90 layers in total: one for the image input, 25 for ConV, 27 for batch normalization (BN), 3 for maximum pooling, 27 for leaky relu (LReLU), 2 for dropout, 3 for FC, 1 for softmax, and 1 for classification. The LReLU AF comes after the ConV layers. The architecture consists of eight fire modules. The Fire module consists of three convolution layers: a squeezing convolution layer with several 1×1-kernel layers, followed by 1×1 and 3×3 convolution layers (expand layer). We selected 1×1 layers to lower the number of parameters. The total amount of parameters in the layer is determined by multiplying the quantity of input channels by the number of kernels and the dimensions of the kernels, i.e., 3. We employed fewer kernels in the squeeze layer than in the expanding layer to decrease the number of inputs (input channels) to filters of size 3×3. To generate the same size output of the 3×3 and 1×1 kernels, we employed one-pixel padding in the 3×3 convolution layers. Each convolutional layer in our architecture is followed by batch normalization and LReLU layers.

**Table 2** reveals the DeepPlantNet framework’s details. In the DeepPlantNet framework, the first (top or initial) layer is an input layer. Its size is comparable to the dimension of the input features, and it comprises I × J units. Our model receives input photos with a length of 227×227-pixel for processing. Convolution layers with a filter of dimensions 3×3 and 1×1 are employed to perform convolutions for the feature map creation. The top or initial convolution layer extracts the feature from the plant leaf pictures (of size 227×227) by employing 64 kernels of 3×3 dimensions with a stride of 2×2. Following the use of ConV and filter, the feature map of the convolution layers (output) is derived as follows:

**Table 2:** DeepPlantNet framework details.

S No	Operation	Layers	No of filters	Filter	Stride	Padding
1		Input				
2	Convolution	Convolution (BN, LReLU)	64	3 × 3	2 × 2	
3	Pooling	Maximum pooling		3 × 3	2 × 2	
4	Fire module	Convolution (BN, LReLU)	16	1 × 1		
		Convolution (BN, LReLU)	64	3 × 3		1 × 1
		Convolution (BN, LReLU)	64	1 × 1		
5	Fire module	Convolution (BN, LReLU)	16	1 × 1		
		Convolution (BN, LReLU)	64	3 × 3		1 × 1
		Convolution (BN, LReLU)	64	1 × 1		
6	Pooling	Max-Pooling		3 × 3	2 × 2	[0 1 0 1]
7	Fire module	Convolution (BN, LReLU)	32	1 × 1		
		Convolution (BN, LReLU)	128	1 × 1		
		Convolution (BN, LReLU)	128	3 × 3		1 × 1
8	Fire module	Convolution (BN, LReLU)	32	1 × 1		
		Convolution (BN, LReLU)	128	3 × 3		1 × 1
		Convolution (BN, LReLU)	128	1 × 1		
9	Pooling	Max-Pooling		3 × 3	2 × 2	[0 1 0 1]
10	Fire module	Convolution (BN, LReLU)	48	1 × 1		
		Convolution (BN, LReLU)	192	1 × 1		
		Convolution (BN, LReLU)	192	3 × 3		1 × 1
11	Fire module	Convolution (BN, LReLU)	48	1 × 1		
		Convolution (BN, LReLU)	192	1 × 1		
		Convolution (BN, LReLU)	192	3 × 3		1 × 1
12	Fire module	Convolution (BN, LReLU)	64	1 × 1		
		Convolution (BN, LReLU)	256	3 × 3		1 × 1
		Convolution (BN, LReLU)	256	1 × 1		
13	Fire module	Convolution (BN, LReLU)	64	1 × 1		
		Convolution (BN, LReLU)	256	3 × 3		1 × 1
		Convolution (BN, LReLU)	256	1 × 1		
14	FC	FC + BN + LReLU + Dropout				
15	FC	FC + BN + LReLU + Dropout				
16	Classification	FC + Softmax + Classification				

The following formula denotes the convolution procedure between the filter and image:


(1) $$f_{c}^{k}\left(m,n\right)=\sum_{d}\sum^{}J_{d}\left(r,s\right).i_{c}^{k}\left(v,w\right)$$

$f_{c}^{k}$ Symbolizes the ultimate feature map, and jd (r, s) symbolizes the plant leaves images which are multiplied by the $i_{c}^{k}$ (v, w) index of the kth filter of the cth layer. After applying convolutions on the plant leaf pictures, the output of size o=((i−f)+2p)/ (s+1) is formed. i stands for input, p for padding, f for kernel size, and s for shift (stride).

The AFs come after convolutional layers. In the past, the sigmoid and tanh AFs were the most common. Most DL applications now use the ReLU and its derivatives (LReLU, Noisy ReLU, and ELU). The weighted sum of the input is converted into output by a node in a layer using the AF. The ReLU deactivates all neurons with values less than 0, making a sizable chunk of the network inactive. Instead of specifying that the ReLU AF be 0 for negative input values, we used an improved ReLU AF (LReLU AF) to define the ReLU as a negligible linear percentage of x. This improved AF improved the model’s classification performance. This AF was determined as follows: In contrast to RelU, the LReLU also produces an output for negative values and does not deactivate the inputs. The following describes how the LReLU AF works:


(2) f(x)=max(0.01×x, x)

The LReLU function returns x when input is positive but 0.01 times x when information is negative (small value).

We used the BN technique to normalize the convolution layer outputs. In addition to enabling regularization, BN speeds up neural network learning and aids in avoiding overfitting. After the first convolutional layer, we used the highest pooling layer with a stride of 2×2 for downsampling. This layer reduces the amount of space, computation, parameters, and calculations.


(3) $$f\left(x\right)=\left\{x_{1},\;x_{2},\;x_{3},\ldots ,\;x_{k}\right\}$$

The best possible feature map is denoted by f(x). In our approach, the upper limit from the nearby pixels (in a plant leaf image) is chosen using maximum pooling, employing a filter of size 3×3 and a stride of length 2×2. After utilizing the AF, BN, LRelU, and max-pooling operation, the output feature of the primary ConV layer is directed to the next ConV layer (in the first fire module).

The ConV layer in the following fire module receives the output of the previous fire module as input. After the second fire module, we used the max-pooling procedure for downsampling. Similarly, following the pooling process, the output of the second fire module is sent to the third fire module. In the convolutional layer, 64 filters of dimension 3×3 with padding of 1×1 are also applied. The additional layer lies before the AF after this ConV layer. We employed the AF after the additional layer. Shortcut connections are used to link every fire module.

The first FC layer accepts the feature map of the last (i.e., 25th) convolution layer. The FC layer transforms the two-dimensional output from the convolution layers into a one-dimensional feature vector. The operations of an FC layer are as follows.


(4) $$a_{i}=\;\sum_{j=0}^{m\times n-1}w_{ij}\times x_{i}+b_{i}$$

where i, n, m, b, d, and w denote the fully connected layer’s output index, height, width, bias, depth, and weights, respectively. The first two FC layers are also followed by BN and LReLU layers. After the first two FC, we utilized the dropout layer (to avoid overfitting). The 8-way softmax (in the event of an eight-class classification) or three-way softmax (in the case of a three-class classification) and classification layers come after the last FC layer. Because of our dataset’s eight classes, the output of the previous FC layer is routed to an 8-way softmax.

### 3.6 Hyper-parameters

The success of DL frameworks depends on the selection of hyper-parameters. We examined the efficacy of the proposed DeepPlantNet framework using a variety of hyper-parameter values in hopes of finding the optimum value for all hyper-parameters given the large variety of options present. To find the best hyperparameters (which provide high accuracy and little error) for the proposed DeepPlantNet framework, we used a grid search approach. We decided to test and evaluate the effectiveness of our DeepPlantNet framework using 25 layers due to the abundance of options for layer numbers, parameters, and kinds. The proposed method’s hyperparameters and additional layers are chosen after some initial tests on a smaller dataset. The actual hyperparameter values are displayed in **Table 3**. We choose the stochastic gradient descent optimization strategy because it is quick, memory-efficient, and works well for more extensive datasets. We trained the algorithm for 35 epochs to account for the potential for overfitting. We employed 80% of the entire data to train DeepPlantNet, and 20% of the images were used to test our model.

**Table 3:** Hyperparameters of the proposed framework.

Parameter	Value
Validation frequency	30
Optimization algorithm	SGDM
Learning rate	0.001
AF	LReLU
Verbose	False
Train Size	0.8
Test Size	0.2
Iterations per epoch	13
Dropout	0.5
Maximum Epochs	35
Shuffle	Every epoch

## 4 Results

This part carefully analyses the results of the many tests conducted to assess how well our framework performs. We describe the experimental approach and performance indicators we employed to evaluate the effectiveness of our approach for classifying plant diseases. This part additionally includes further details on the dataset. To assess the effectiveness of our plan, we used a Kaggle dataset available to the general audience.

### 4.1 Performance evaluation on plant disease classification (eight disease categories)

**Figure 3:**
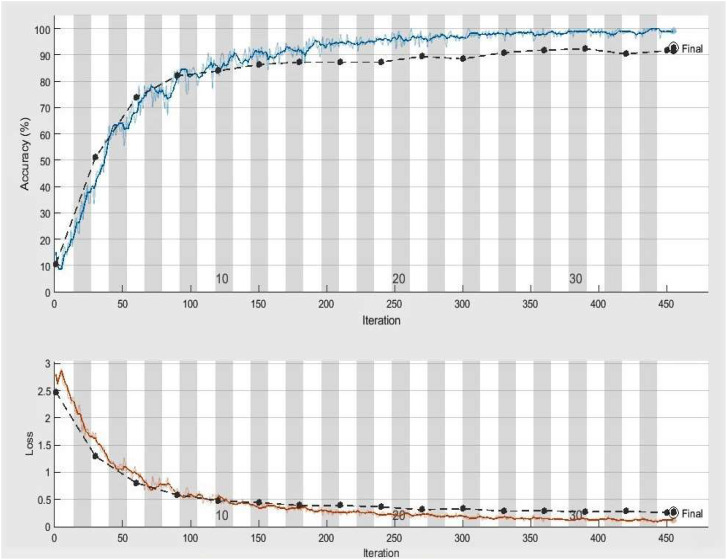
Accuracy and loss achieved by DeepPlantNet framework (eight class classification), the black line demonstrates the testing and training accuracy and loss, whereas the blue and red lines display training accuracy and activity loss, respectively.

The significant goal of this experiment is to confirm the usefulness and worth of our approach in identifying and classifying plant diseases. In this experiment, we employed 2080, more precisely 260 infected leaf photos of eight species (apple, cherry, grape, peach, pepper, potato, squash, and strawberry) from the PlantVillage dataset. We have used images of one disease from each species, i.e., ABR, CPM, GLB, PBS, PBBS, PEB, SPM, and SLS. A total of 1664 images of plant diseases were utilized in training, whereas 416 images were utilized for testing our framework. On the similar experimental conditions shown in **Table 3** for classifying eight categories of infected leaves illnesses photos, we trained our DeepPlamtNet model using the training set. Our DeepPlantNet model needed 2550 minutes and 56 seconds to train for plant disease identification. The loss function demonstrates how well our approach can forecast our dataset. We illustrated accuracy and loss in **Figure 3** to illustrate the training performance of the proposed strategy, illustrating that we may get acceptable accuracy even at lesser classification epochs. The suggested framework successfully classified plant diseases into many classes, achieving the ideal average accuracy, recall, precision, and F1-score of 98.49%, 94.125%, 93.87%, and 94.00%. To precisely describe the classification performance of the suggested technique in terms of actual and expected classes, we additionally built a confusion matrix analysis.

**Table 4** displays the proposed confusion matrix for the DeepPlantNet method. The confusion matrix is a tabular representation of our DeepPantNet model’s performance. Each entry in a confusion matrix indicates the number of predictions provided by the DeepPlantNet model that were accurately or inaccurately categorized by the classes. According to the confusion matrix (**Table 4**), the DeepPlantNet model can correctly classify the eight plant diseases (ABR, CPM, GLB, PBS, PBS, PEB, SPM, and SLS), as shown in **Table 4**. All 255 out of 260 GLB images are accurately identified by the proposed model, followed by PEB (251 out of 260 images of each disease are correctly classified by the proposed model). Nevertheless, the model accurately categorized only 239 out of 260 PBS photos, which fared worse when forecasting PBS illness. As the proposed framework correctly classified most of the disease categories image samples (**Table 4**), according to the confusion matrix, our technique attains the best outcomes with a high TP rate for all the plant disease categories in our dataset.

**Table 4:** Confusion matrix obtained by DeepPlantNet framework for eight class classifications.

Predicted Class									
True class	Plant disease	ABR	CPM	GLB	PBS	PBBS	PEB	SPM	SLS
	ABR	48	0	0	1	3	0	0	0
	CPM	2	47	0	1	1	0	1	0
	GLB	0	0	52	0	0	0	0	0
	PBS	1	0	0	47	3	0	0	1
	PBBS	4	1	1	1	40	5	0	0
	PEB	1	0	1	0	2	47	0	1
	SPM	0	0	0	1	0	0	50	1
	SLS	0	0	0	0	0	0	2	50

Accurate identification, detection, or classification of unhealthy plant leaves is necessary to determine the efficacy and validity of the suggested approach. To achieve this, we evaluate the DeepPlantNet approach’s usefulness in classifying each disease (class-wise performance). **Table 5** displays the suggested DeepPlantNet approach’s performance in terms of precision, accuracy, F1-score, and recall for classifying plant leaf diseases. According to **Table 5**, the proposed method offers cutting-edge performance in every evaluation criterion. According to the results, most photographs are accurately categorized, increasing accuracy. The essential factor for the increased plant illness identification accuracy is the robustness of the newly proposed framework, which more accurately reflects each class.

**Table 5:** Class-wise performance of the DeepPlantNet framework in the case of eight class classifications.

Class	N (classified)	N (truth)	Accuracy	Recall	Precision	F1 score
ABR	265	260	98.41	95.0	93.0	94.0
CPM	258	260	98.75	95.0	95.0	95.0
GLB	267	260	99.18	98.0	96.0	97.0
PBS	256	260	98.17	92.0	93.0	92.5
PBBS	252	260	97.02	87.0	89.0	77.5
PEB	263	260	98.99	97.0	95.0	96.0
SPM	261	260	98.8	95.0	95.0	95.0
SLS	258	260	98.65	94.0	95.0	94.5

**Figure 4:**
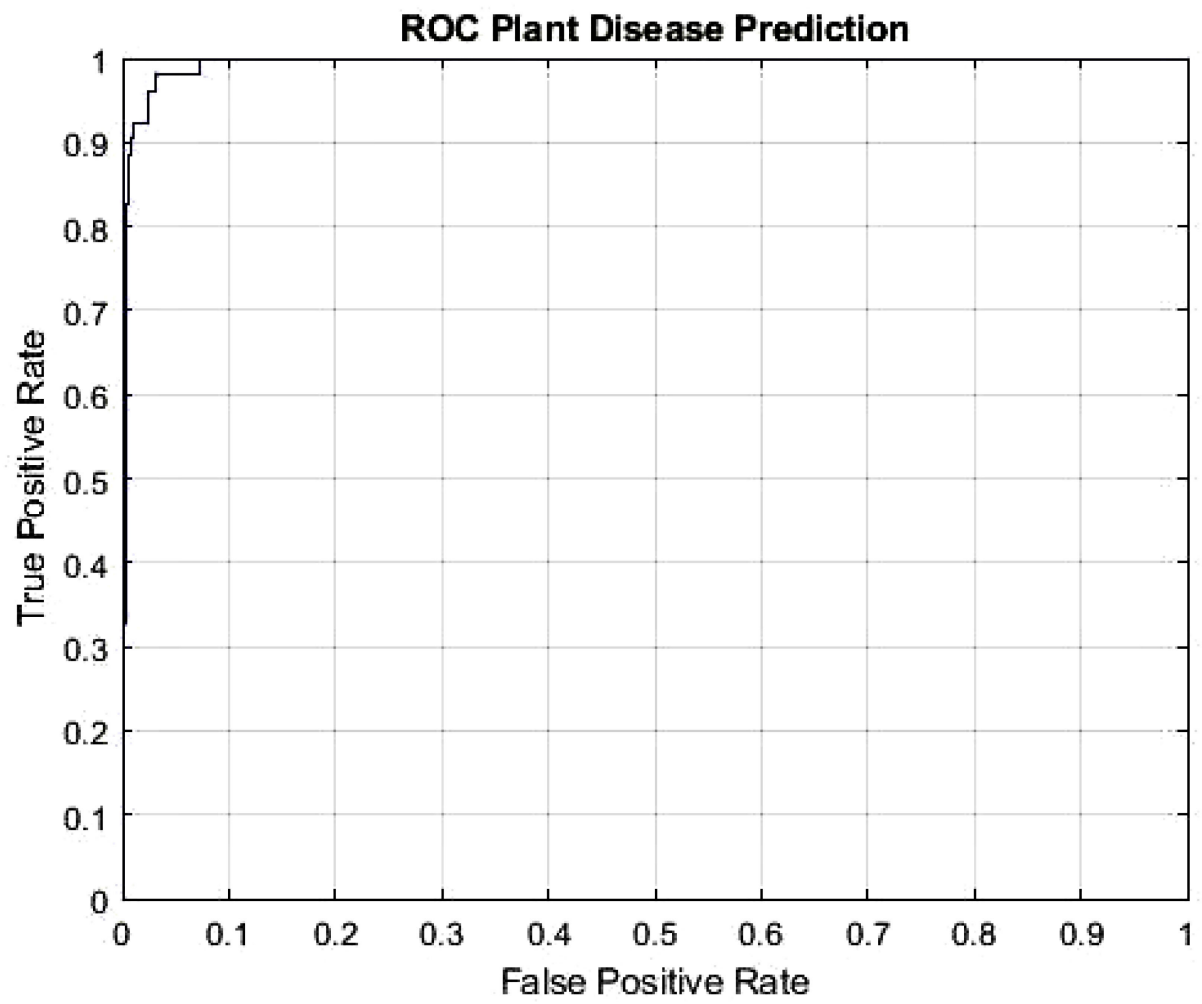
ROC plot of the DeepPlantNet model (eight class classification).

The DeepPlantNet framework’s Receiver Operating Characteristic (ROC) curve is depicted in **Figure 4** and demonstrates how effectively it detects plant diseases. To determine the ROC, we utilized the MATLAB function per curve. The ROC uses threshold values to outputs in the [0,1] range. The FP Ratio and TP Ratio are computed for each threshold. The TP to FP ratio is shown on the ROC curve, demonstrating the algorithm’s sensitivity. The area under the curve (AUC) measures how distinct classes vary from one another, making it a key assessment criterion for algorithms. It determines how effectively the algorithm can distinguish between classes. The framework will be more effective at differentiating between various disease classes if the AUC value is close to 1. Our model’s AUC score was 0.9926.

Our model includes convolutional layers with kernels of varying sizes (3 × 3, 1 × 1), so the proposed DeepPlantNet technique provides the best classification accuracy. It enables the network to pick up on distinct spatial patterns and identify traits at different scales. Patterns are found via 1×1 kernels through the depth of the input photos. At the same time, 3 × 3 filters discover spatial patterns across the three dimensions of the input (width, depth, and height). As a result, diverse convolutional kernel sizes learn varied spatial patterns at varying scales and more accurately extract distinguishing information from plant leaf images.

### 4.2 Performance evaluation on plant disease classification (three disease categories)

**Figure 5:**
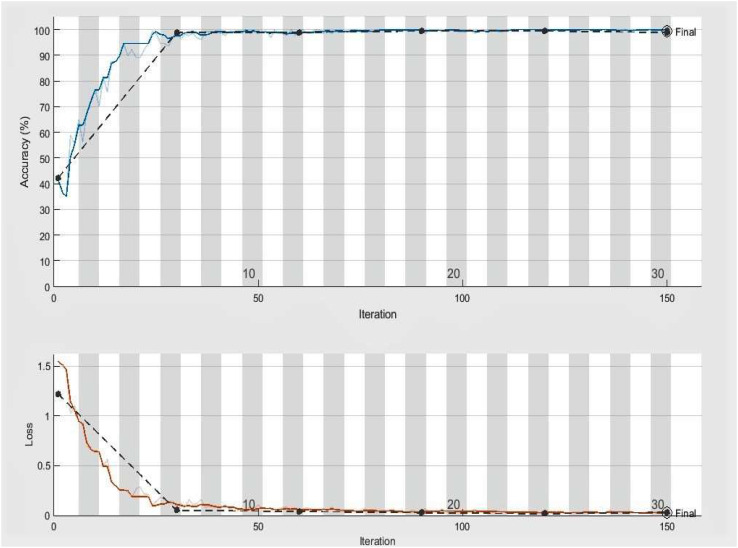
Accuracy and loss achieved by the proposed model (three class classification), the black line displays the testing and training accuracy and loss, whereas the red and blue lines show training loss and training accuracy, respectively.

The significant objective of this experiment is to confirm the effectiveness and usefulness of the presented approach in identifying and classifying plant diseases (three categories). We validated our model using another common, publicly accessible Kaggle dataset, “Plant Disease Prediction Dataset,” to assess and estimate the generalizability and performance of the DeepPlantNet model (Dataset: https://www.kaggle.com/datasets/shuvranshu/plant-disease-prediction-dataset). The data includes three plant diseases: bacterial tomato spot, an early blight on potatoes, and common corn (maize) rust. The dataset has 300 infected leaves photos from each class for model training and is balanced. In this experiment, we employed 900, more precisely 300, infected leaf photos of three different species (maize, potatoes, and tomato) from the “Plant Disease Prediction Dataset” dataset. Seven hundred twenty images of plant diseases are utilized for framework training, and the outstanding 180 pictures are utilized for framework testing. On the undistinguishable experimental conditions listed in **Table 3** (except the number of epochs) for the classification of three categories of infected leaves illnesses photos, we trained our DeepPlantNet model using the training set. Our DeepPlantNet model needed 727 minutes and 4 seconds to train for plant disease identification. We illustrated accuracy and loss in **Figure 5** to illustrate the training performance of the proposed strategy, illustrating that we may get acceptable performance even at more minor classification epochs (i.e., epochs 6). The testing accuracy and loss almost remain the same after epoch 6. The suggested method successfully classified plant diseases into three classes, achieving the perfect average precision, accuracy, F1-score, and recall of 99.66%, 99.85%, 99.82%, and 100%. To precisely describe the classification performance of the suggested method in terms of actual and expected classes, we additionally built a confusion matrix analysis. **Table 6** displays the proposed confusion matrix for the DeepPlantNet method—a confusion Matrix is employed to evaluate the accuracy of the prediction. The confusion matrix provides a comparison between predicted and actual values. The proposed DeepPlantNet framework successfully classified all images (60 out of 60 images) of bacterial tomato spot and common maize rust correctly, whereas misclassified only one image of the early potato blight disease as bacterial tomato spot (classified 59 potato early blight disease images correctly), according to the confusion matrix, which shows that the suggested method attains the best performance with a high TP rate for all the plant disease categories in our dataset.

**Table 6:** Confusion matrix achieved by the proposed model in case of three class classification.

Predicted Class				
True Class	Disease class	Maize common rust	Potato early early blight	Tomato bacterial spot
	Maize common rust	299	0	0
	Potato early early blight	0	299	0
	Tomato bacterial spot	1	1	300

**Figure 6:**
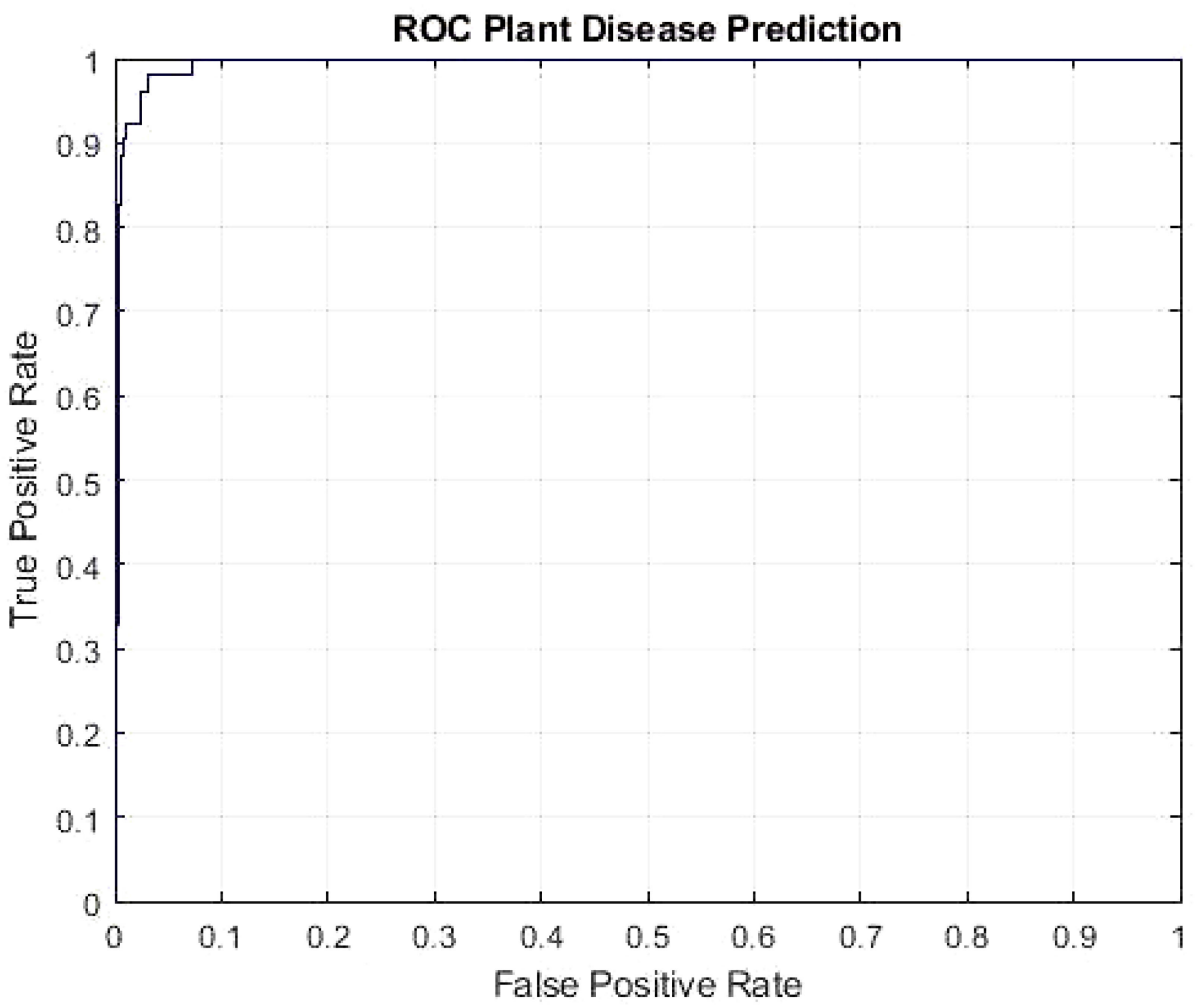
ROC plot of the proposed DeepPlantNet framework (three-class classification).

According to the results, most photographs are accurately categorized, increasing accuracy. The essential factor for the expanded plant disease classification accuracy is the robustness of the newly suggested framework, which more accurately reflects each class. The ROC AUC of our framework is shown in **Figure 6**. Our model is more effective at differentiating between various disease classes and achieved the AUC score of 0.9950.

### 4.3 Performance evaluation on plant disease classification (six disease categories)

To further validate the effectiveness of our model we used both normal and infacted leaf images of three types of plants i.e., tomato, potato, and maize. The objective of this experiment is to check the validity and usefulness of the proposed approach in detecting and classifying plant three types of leaf diseases (three categories) as well as normal images of those particular plants. We validated our model using common, publicly accessible PlantVillage to assess and estimate the generalizability and performance of the DeepPlantNet model in the presence of normal and infected plant leaves. The data includes three plant diseases: bacterial tomato spot, an early blight on potatoes, and common corn (maize) rust and normal images of these three plants i.e., tomato, potato, and maize. In this experiment, we used 2906 leaf images of healthy plants (1592 images of tomatoes, 1162 images of maize, and 152 images of potatoes). And 900 leaf images of infected plants, more precisely 300, infected leaf photos of three different species (maize, potatoes, and tomato. We used 5-fold cross validation in which 20% of the data is used for the testing and 80% of the data is used for training of our model in each fold. On the undistinguishable experimental conditions listed in **Table 2** for the classification of three categories of infected leaves illnesses photos, we trained our DeepPlantNet model using the training set. Our DeepPlantNet model needed 890 minutes and 8 seconds to train for plant leaf disease identification. The suggested method successfully classified plant diseases into three classes, achieving the perfect average accuracy of 99.85% and all precision, F1-score, and recall of 99.00%. To precisely describe the classification performance of the suggested method in terms of actual and expected classes, we additionally built a confusion matrix analysis. **Table 7** displays the proposed confusion matrix for the DeepPlantNet method—a confusion Matrix is employed to evaluate the accuracy of the prediction. The proposed DeepPlantNet framework successfully classified most of the images (299 out of 300 images) of common maize rust correctly, whereas misclassified eight images of the early potato blight disease, according to the confusion matrix, which shows that the suggested method attains the best performance with a high TP rate for the three plant disease categories and healthy images in our dataset.

**Table 7:** Confusion matrix achieved by the proposed model in case of six class classification.

Predicted Class							
True Class	Disease class	Maize common rust	Maize healthy	Potato early blight	Potato healthy	Tomato bacterial spot	Tomato healthy
	Maize common rust	299	0	0	0	0	0
	Maize healthy	1	1162	1	0	0	0
	Potato early blight	0	0	292	0	1	1
	Potato healthy	0	0	4	150	2	0
	Tomato bacterial spot	0	0	2	2	295	0
	Tomato healthy	0	0	1	0	2	1590

### 4.4 Comparison with state-of-the-art deep learning models

This experiment evaluates the usefulness and success of the suggested DeepPlantNet framework for plant disease classification into eight classes over the different current DL-based frameworks. We used the dataset with eight types of plant diseases for comparison because we have achieved minimum results in this case. Therefore, we compared our proposed DeepPlantNet framework performance with these comparative models, i.e., ResNet18 (He et al., 2016), Denenet201 (Huang et al., 2017), Darknet19 (Redmon, 2016) and MobileNetv2 (Sandler et al., 2018, Ullah et al., 2023a). Using a transfer learning technique, these modern DL frameworks were trained on millions of photos from the ImageNet database. All pre-trained variants of the networks’ final layer were fine-tuned to divide the pictures into eight classes: ABR, CPM, GLB, PBS, PBBS, PEB, SPM, and SLS. Network input picture sizes vary depending on the network. For example, darknet19’s input image size is 227 by 227, whereas resnet18’s is 224 by 224. We resized the input image size to meet the requirements of each contrasting DL framework. For this test, 1664 pictures of plant diseases are utilized for the training phase, and the leftover 416 pictures are utilized for testing our framework. The DeepPlantNet framework is trained using the training set and the same experimental settings for classifying plant diseases as those listed in **Table 2**. **Table 8** presents the findings. These outcomes demonstrate the efficacy of our suggested approach for classifying plant diseases into ABR, CPM, GLB, PBS, PBBS, PEB, SPM, and SLS. Our model obtained an accuracy of 97.89%, a precision value of 91.37%, a recall value of 91.5%, and an F-measure value of 91.43%, which is superior to all other four contemporary models in terms of all performance measures. The resenet18 framework had the lowest accuracy of all the models (95.24%), while the densenet201 model had the second-best accuracy (96.12%). These comparative outcomes prove our approach’s superiority over comparative frameworks for plant disease classification into ABR, CPM, GLB, PBS, PBBS, PEB, SPM, and SLS.

**Table 8:** Plant disease classification into ABR, CPM, GLB, PBS, PBBS, PEB, SPM, and SLS comparison with state-of-the-art frameworks.

Model	Precision	Accuracy	Recall	F1-score
Resnet18	89.66	95.24	91.33	90.48
Mobilenetv2	91.0	97.34	91.0	91.0
Densenet201	90.66	96.12	90.0	90.33
Darknet19	90.0	95.47	91.11	90.49
DeepPlantNet	91.37	97.89	91.5	91.43

## 5 Discussion

Most researchers used ML-based approaches for plant disease classification in the past. But the critical drawback of using ML-based approaches for classifying plant diseases includes the lengthy codes that increase computer complexity, low efficiency, and prolonged processing times. Several approaches have been created to handle the problem of long codes. The trade-off is that the code is now more complex. Some recent publications propose DL-based algorithms and models for classifying plant leaf diseases, inspired by the enormous achievement of DL-based algorithms. This paper has created our new DL-based DeepPlantNet framework for plant disease classification. Our approach categorizes ten types of plant diseases. We have performed two experiments, i.e., eight-class classification and three-class classification of plant leaf diseases. The proposed model identified accurately and reliably plant diseases by achieving an average accuracy of 98.49and 99.85in the case of eight-class and three-class classification schemes, respectively. Our approach achieved remarkable performance even by using limited images of each class (250 images per class) for training and testing. Modern DL frameworks that may be discovered in the literature are compared to the suggested model.

We performed experiments on small dataset to find the number of layers in our model which can provide optimal results. Our study technique works well since the proposed DeepPlantNet framework incorporates the LReLU AF (Ullah et al., 2023b) rather than the ReLu AF. To overcome the issue of dying ReLU, we employed the LReLU AF. The DL network won’t function if ReLU (Ullah et al., 2022b) fails for whatever reason. We added an LReLU to the proposed DeepPlantNet method to fix this problem. The LReLU activation method permits a modest (non-zero) gradient when the unit is unused. As a result, it keeps learning rather than stopping or hitting a brick wall. Consequently, the LReLU AF’s increased feature extraction capabilities enhance the proposed DeepPlantNet model’s effectiveness in identifying plant illnesses. These results are also because we have used enough layers (25 convolutional layers) in our proposed technique which can successfully extract the most distinctive, robust, and detailed features to represent the plant leaf images for accurate and effective classification. The initial convolutional layers extract (low-level) characteristics like color, edges, etc. Deeper layers, in comparison, oversee extracting high-level information like an anomaly in the images of plant leaves. Moreover, BN is utilized to speed up training, standardize the inputs, stabilize the network, lessen the number of epochs, and offer regularization to stop the network from overfitting (Ullah et al., 2022c). Also, we used small filters of size 1×1 and 3×3 to extract more detailed features. The size of the feature maps is reduced by pooling layers. The fundamental benefit of pooling is the extraction of clean, angular features. Additionally, it is done to cut down on calculations, parameters, and variation. In order to extract low-level features like points, edges, etc., max-pooling is helpful. Moreover, we have used dropout layer to overfitting on training data.

Furthermore, an experiment was created to evaluate the DeepPlantNet model’s effectiveness in classifying plant diseases compared to other cutting-edge approaches. Because of variations in data pre-treatment, training and validation methods, datasets, type of plant diseases classified, and processing power used in the respective methodology, this is not a direct comparison. In (Bensaadi and Louchene, 2023), the authors presented a low-complexity CNN-based framework for automatic plant disease classification that allows for quicker online categorization. More than 57,000 tomato leaf pictures were used in the training procedure. The images of tomato leaves from nine classes captured in a natural setting were used in training without background subtraction. The developed model successfully classified the diseases with an accuracy of 97.04% and less than 0.2 errors, demonstrating its remarkable precision. A computerized technique was suggested for accurately recognizing and categorizing illnesses from a given image (Haridasan et al., 2023). The suggested scheme for identifying rice plant illnesses employs a computer vision-based method that uses the techniques of image processing, ML, and DL to safeguard rice plants against the five major diseases that regularly affect the Indian rice fields. This lessens the need for traditional techniques. Image segmentation was used to pinpoint the paddy plant’s affected region after image pre-processing. The aforementioned ailments can be identified only by their outward symptoms. An SVM and CNN were used to identify and categorize specific types of paddy plant illnesses. Using ReLU and softmax algorithms, the suggested DL-based approach achieved a maximum validation accuracy of 0.9145. Following diagnosis, a preventative strategy was put out to assist those involved in agriculture and organizations in effectively combating these illnesses. The authors (Nagi and Tripathy, 2023) developed a fuzzy feature extraction method based on probabilistic neural networks (PNN) recognition capability to identify plant leaf disease. They first extracted texture and color information from leaf images using fuzzy gray-level co-occurrence matrices and fuzzy color histograms and then used PNN for classification. The PlantVillage database obtained the tomato, grapevine, and corn leaf photos. The suggested model beats existing classifiers like RF, DT, and SVM and receives a recognition accuracy of 95.68%. Bi-linear CNNs were employed by the authors (Srinivasa Rao et al., 2022) for the detection and categorization of plant leaf diseases.

The model’s accuracy for 38 different classes was 94.98% when tested against industry-recognized categorization metrics. A novel DenseNet with multilayer perceptron (MLP)-based diagnostic for rice plant disease, DenseNet169-MLP, was created (Narmadha et al., 2022). The suggested model sought to categorize the three rice plant diseases, i.e., Brown Spot, Leaf Smut, and Bacterial Leaf Blight. The pre-trained DenseNet169 was employed as a feature extractor to accomplish rice plant disease identification, and the MLP was used in place of the final layer. With a high accuracy of 97.68%, the findings showed that the DenseNet169-MLP model performed better than the recently described approaches. A novel method for rapidly recognizing and classifying plant leaf diseases was proposed using the ELM DL algorithm on an actual database of plant leaf images (Aqel et al., 2022). For image segmentation, feature extraction, feature selection, and classification of plant leaf diseases, the suggested method employed the k-means clustering, GLCM, BDA optimization, and ELM algorithms, respectively. Seventy-three plant leaf photos from four disease classes—Alternaria alternata, Anthracnose, Bacterial blight, and Cercospora leaf spot—were included in the dataset utilized for this investigation. The testing findings demonstrated that the proposed technique had a heartening categorization accuracy of 94%. The outcomes of this experiment (comparison study) show that the proposed plant disease classification system is workable.

Regarding average accuracy, we discovered that the suggested framework worked reasonably well, obtaining the highest overall accuracy of 97.89% and 99.62% in the case of eight-class and we three-class categorization arrangements (**Table 9**). Notably, these models were computationally challenging and demanded considerable processing power and hardware. However, because of the end-to-end learning framework utilized in the suggested DeepPlantNet technique, our solution does not require further feature extraction, selection, or segmentation steps. This study suggested an automated system for the classification of plant leaf diseases. The proposed DeepPlantNet model is lightweight and has less parameters than the techniques utilized in the literature, which are based on CNN models that employ a lot of deep layers and many parameters.

**Table 9:** Comparison with state-of-the-art approaches.

Work	Method	Classification scheme	Sample size	Accuracy (%)	Date
Bensaadi and Louchene (2023)	Low-cost CNN	Nine-class	57,000	97.04	2023
Haridasan et al. (2023)	CNN + SVM	Five-class		91.45	2023
Nagi and Tripathy (2023)	Fuzzy feature and PNN	17-class		95.68	2023
Srinivasa Rao et al. (2022)	bi-linear convolution neural network	38-class	54,305	94.98%	2023
Narmadha et al. (2022)	DenseNet169-MLP	3-class	120	97.68%	2022
Aqel et al. (2022)	Extreme learning machine	4-class	73	94%	2022
This study	DeepPlantNet framework	Eight-class	2080 and 900	97.89% and 99.62%	2023

Although the proposed approach produced positive results, we identified shortcomings and offered suggestions for further investigation. The PlantVillage dataset is utilized to assess how well our DL model achieved. Despite many images of various plant species with their diseases in this dataset, the images were all taken in a lab. Consequently, a substantial dataset of plant diseases in actual environments is expected to be created. Because plant diseases can vary in intensity over time, DL algorithms should be enhanced or updated to recognize and categorize illnesses during their whole life cycle. Since the DL architecture should function well in various illumination conditions, the datasets should include images taken in varied field situations. It is essential to properly examine all the factors that may affect the discovery of plant illnesses, including the kinds and sizes of databases, learning rates, brightness, and other elements. Unfortunately, this study only investigates significant plant diseases. We want to enlarge the categorization size for the proposed DeepPlantNet framework to identify plant illnesses correctly. Moreover, we have trained our model on limited number of images which is not enough to capture the diversity and variability of plant diseases. So, in future we will validate the generalization ability of our proposed method by training it with large scale datasets both in agricultural and medical domains (Yousaf et al., 2023; Raza et al., 2023). This study may aid professionals and farmers in making more rapid and accurate plant disease identifications, minimizing financial and crop yield losses. Furthermore, a similar technique may be applied to identify and classify numerous diseases in other plants and categorize the different types of disease utilizing other parts of plants (such as stem or flowers, etc.).

## 6 Conclusion

Agriculture is essential to a nation’s economic development. However, plant disease is the main danger to agricultural output and quality. Early detection of plant diseases is crucial for the health and welfare of the entire world. Unfortunately, accuracy issues and a lack of personnel resources limit manual crop disease inspection. Automated approaches for identifying and categorizing plant diseases are needed to solve these problems. A DeepPlantNet framework for efficient plant disease detection and classification was provided in this paper. The suggested framework’s superiority over existing techniques has been demonstrated by the average accuracy of 97.89% and 99.62% for eight-class and three-class classification schemes, respectively, for plant disease recognition and classification. Additionally, experimental findings on datasets related to plant diseases have validated the efficacy and reliability of the suggested framework for detecting and classifying plant illnesses.

## Data availability statement

The original contributions presented in the study are included in the article/supplementary material. Further inquiries can be directed to the corresponding authors.

## Author contributions

NU Developed the method, detailed investigation and manuscript writing, JK, NR, SE-S, and SA revised the methodology, supervised and revised the manuscript writing (revised draft). MA, FA, and MQ revised the experiments and analysis and. edited the manuscript where necessary (Final draft). All authors contributed to the article and approved the submitted version.
